# Comparison of Efficacy and Safety of Endoscopic Retrograde Cholangiopancreatography in Choledocholithiasis Patients at Different Age Groups: A Meta-Analysis

**DOI:** 10.5152/tjg.2025.24003

**Published:** 2025-01-13

**Authors:** Bo Wang, Jie Cheng

**Affiliations:** 1 Department of General Surgery, Xijing 986 Hospital, Air Force Medical University, Xi’an, China; 2 Department of Maternity, Xijing 986 Hospital, Air Force Medical University, Xi’an, China

**Keywords:** Choledocholithiasis, complete stone clearance, complication, endoscopic retrograde cholangiopancreatography

## Abstract

**Background/Aims:**

Endoscopic retrograde cholangiopancreatography (ERCP) is often recommended as the first choice for the treatment of choledocholithiasis in the elderly. This study aims to investigate the efficiency and safety of ERCP in choledocholithiasis patients of different age groups.

**Materials and Methods:**

Study searching was performed in the PubMed, Embase, Web of Science, and Cochrane Library databases from the inception to August 2024. The outcomes were complete stone clearance, mortality, overall complications, pancreatitis, perforation, biliary infection, bleeding, and pneumonia. Choledocholithiasis patients were divided into young (<65 years), general old (65 years ≤ age <80 years or 65 years ≤ age <90 years), and extremely old (≥80 years or ≥90 years) groups.

**Results:**

Finally, 10 eligible studies were included for analysis. Compared to extremely old patients (≥90 years), the complete stone clearance was higher [odds ratio (OR) = 7.60, 95% CI: 1.89-30.57] and pneumonia was lower (OR = 0.16, 95% CI: 0.06-0.41) in general old patients (65 years ≤ age <90 years). Young (<65 years) patients had lower odds of mortality when compared to the age ≥65 years group (OR = 0.21, 95% CI: 0.17-0.27) and the age ≥80 years group (OR = 0.19, 95% CI: 0.15-0.24). In the comparison of 65-80 years versus ≥80 years, lower mortality (OR = 0.80, 95% CI: 0.65-0.98) was observed in the group of age range 65-80 years.

**Conclusion:**

Our findings suggested that extremely old patients with choledocholithiasis should cautiously choose ERCP, and postoperative complications should be monitored in extremely old patients.

**Main Points**

- Complete stone clearance was lower in extremely old patients.
- Mortality was higher in extremely old patients.
- Extremely old patients had higher odds of pneumonia.
- Endoscopic retrograde cholangiopancreatography should be cautiously used for extremely old patients with choledocholithiasis.

## Introduction

Choledocholithiasis refers to the presence or formation of gallstones in the common bile duct (CBD).^1^ Cholelithiasis is very common among the general population (the incidence ranges from 5% to 15%), with 5%-15% accompanied by choledocholithiasis.^2^ In addition, the incidence of choledocholithiasis increases with age.^3^ Evidence has shown that biliary surgeries are related to an elevated risk of morbidity and mortality.^2^ Therefore, there is a tendency to apply more conservative treatment in very elderly patients.

Endoscopic retrograde cholangiopancreatography (ERCP) is an important non-surgical treatment and is often recommended as the first choice for the treatment of choledocholithiasis in the elderly.^1^ Although ERCP is an effective procedure for removing stones under endoscopy, it causes a high risk of complications, such as pancreatitis, cholangitis, and bleeding.^4^ Due to the impact of the aging population, the number of elderly patients receiving treatment for bile duct stones is expected to increase.^4^ The comorbidities index of elderly patients is higher than that of younger patients.^5^ With the increase in comorbidities, mortality is increased in the older population, and the odds of postoperative death are 10 times higher in elderly patients.^6^ A meta-analysis by Iqbal et al^7^ found no statistical significance in the technical success rate and risk of adverse events in patients ≥80 years compared to younger patients. The elderly are defined as people older than 65 years, which is a wide age range and is heterogeneous in many aspects.^8^ The physical condition of people in their 60s greatly differs from that of people in their 80s.^9^ Therefore, there is a need to explore the efficacy and safety of ERCP in different age groups.

This meta-analysis aims to explore the efficacy and safety of ERCP in choledocholithiasis patients at the young stage (<65 years), general old stage (65 years ≤ age <80 years or 65 years ≤ age <90 years), and extremely old stage (≥80 years or ≥90 years) based on currently available studies.

## Materials and Methods

### Literature Search Strategy

This meta-analysis was performed according to preferred reporting items for systematic reviews and meta-analyses guidelines. Two independent researchers (B.W. and J.C.) searched the PubMed, Embase, Web of Science, and Cochrane Library databases from the inception to August 2024 for relevant published articles. The search strategies are shown in Supplementary File 1. Disagreements between the 2 researchers were resolved through consensus.

### Inclusion and Exclusion Criteria

Inclusion criteria: (1) reporting choledocholithiasis patients undergoing ERCP; (2) study population divided into 2 or 3 of the following groups: young (<65 years), general old (65 years ≤ age <80 years or 65 years ≤ age <90 years), and extremely old (≥80 years or ≥90 years); (3) reporting outcomes: complete stone clearance, mortality, complications (overall complications, pancreatitis, biliary infection, bleeding, perforation, pneumonia); and (4) study design: cohort study or case–control study.

Exclusion criteria: (1) case reports, conference abstracts, letters, reviews, and meta-analyses; (2) unable to extract the data; and (3) not published in English.

### Data Extraction

Data were extracted by 2 researchers (B.W. and J.C.) as follows: the first author, publication year, country, study design, population, group, sample size, sex, diabetes mellitus, cardiovascular diseases, and endoscopic sphincterotomy. Any disagreements between the 2 researchers were resolved through consensus. Information on informed consent and ethics committee approval is not applicable to meta-analysis.

### Quality Assessment

Newcastle Ottawa scale (NOS) was applied for the assessment of the quality of cohort studies and case–control studies, which contained 3 items: selection of study population, comparability of the groups, and outcome evaluation (cohort)/exposure (case–control).^10^ The total score of this scale was 9, and assessed the study as low (1-3 points), moderate (4-6 points), and high quality (7-9 points).

### Statistical Analysis

All statistical analyses were performed using Stata 16.0 (StataCorp, College Station, TX, USA). The counting data were presented as odds ratio (OR), with a 95% CI. The *I*^2^ statistic was used to assess the variance attributable to heterogeneity between individual studies. If *I*^2^ < 50%, a fixed-effect model was used for analysis. If *I*^2^ ≥ 50%, a random-effect model was used for analysis. Publication bias was not assessed because the studies included for each outcome were not more than 10 studies. Sensitivity analysis was performed to assess the robustness of the results by successively excluding the studies. *P* < .05 was regarded as the statistical significance.

## Results

### The Selection and Characteristics of Patients

**Figure 1.**
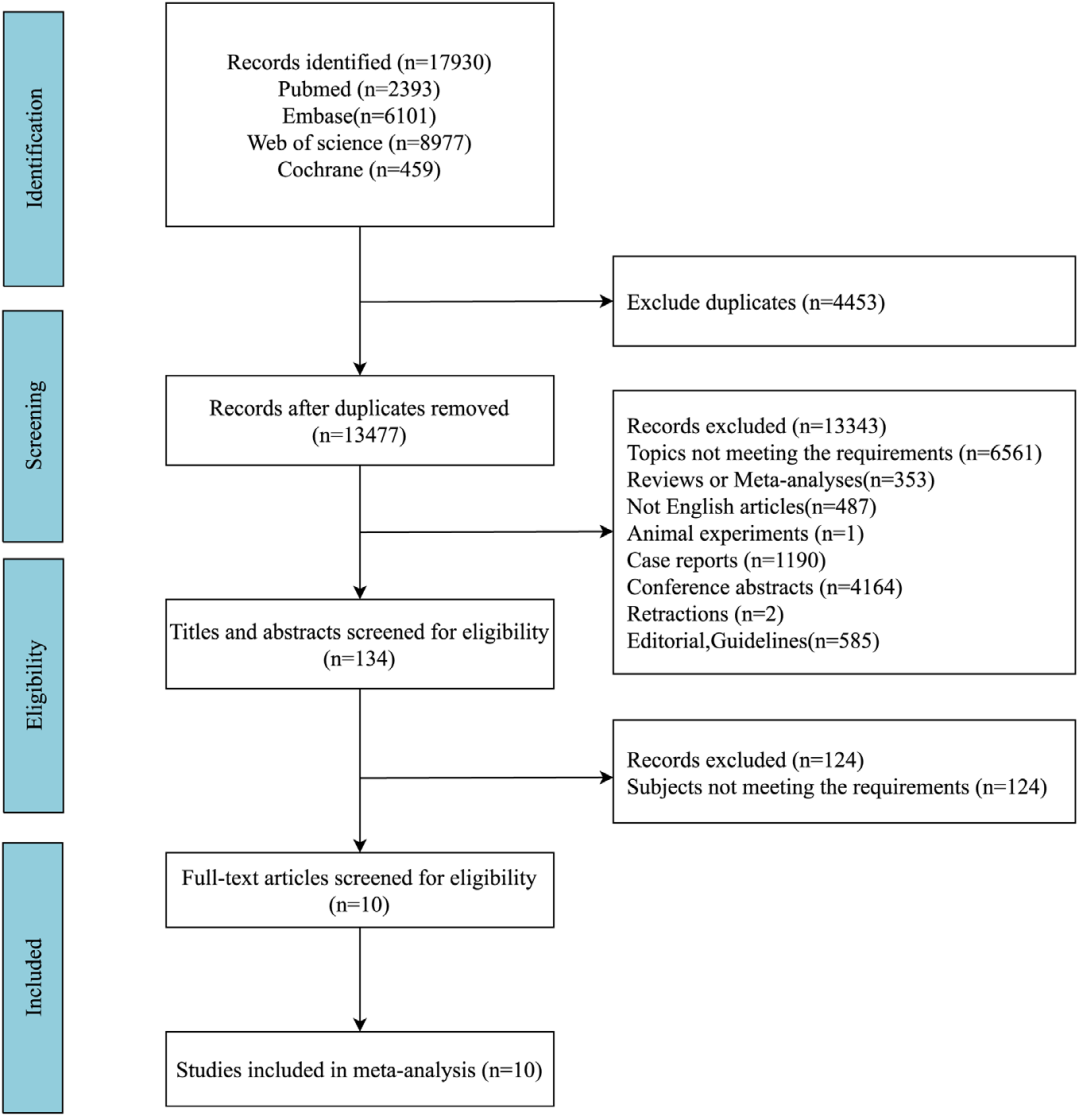
The flowchart of studies selection.

The initial search revealed 17 930 articles; of these, 4453 duplicates were excluded. After screening titles and abstracts, 1190 case reports, 4164 conference abstracts, and 6561 studies not meeting the topic were excluded. After screening the full texts, 10 eligible studies (supplementary material of included literature) were included (Figure 1). Table 1 provides the details of characteristics and NOS scores of included studies. The 10 studies were performed in America, China, Japan, Europe, Sweden, and the United Kingdom. Nine of these were cohort studies, and 1 was a case–control study. According to the NOS score, 4 studies were of high quality, and 6 studies were of moderate quality.

### Comparison of Complete Stone Clearance in Choledocholithiasis Patients at Different Age Groups

**Figure 2.**
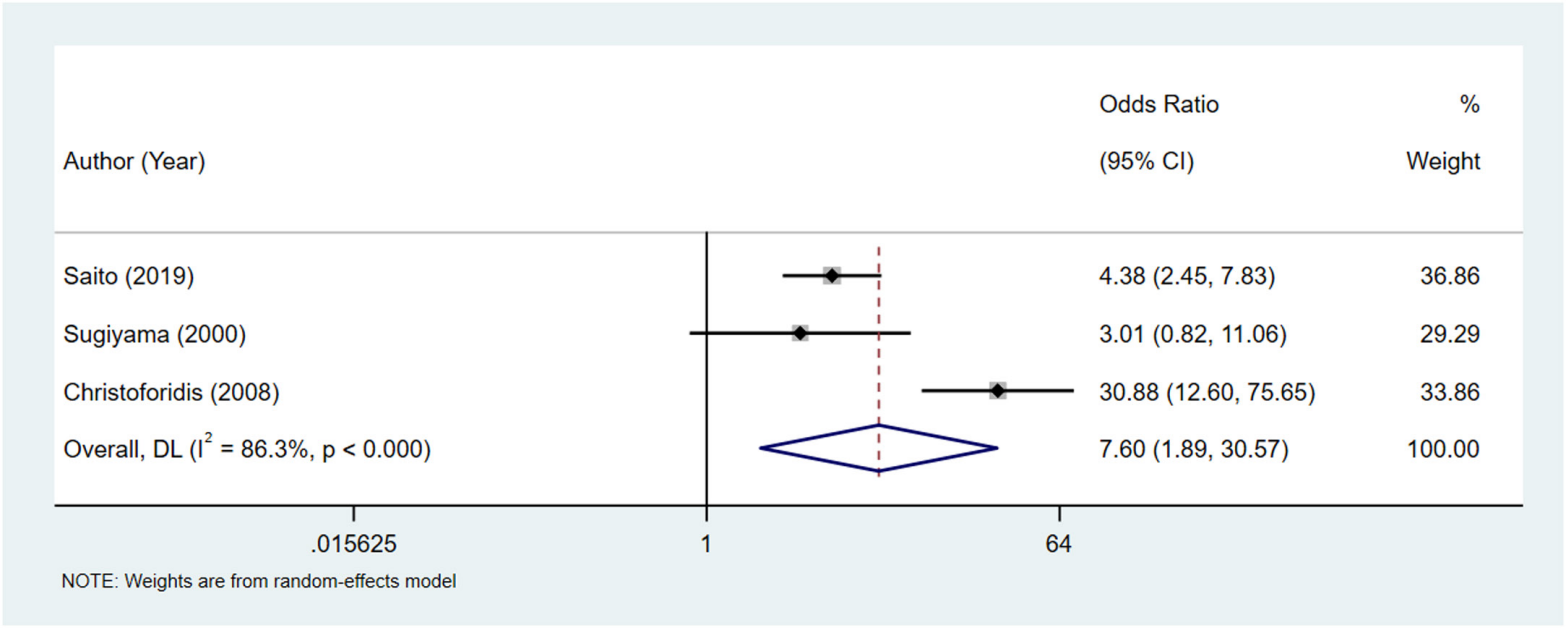
Forest plot regarding to the complete stone clearance between the age range 65-90 vs. age ≥90.

Table 2 shows that one study compared the complete stone clearance between different age groups. When comparing the age <65 years group to the age ≥65 years group, results showed no statistical significance (*P* = .697). Two studies compared the complete stone clearance between the age <65 years group and the age ≥80 years group, and no difference was found in complete stone clearance (*P* = .474). The pooled results displayed that the complete stone clearance was higher in the age range 65-90 years group than the age ≥90 years group (OR = 7.60, 95% CI: 1.89-30.57) (Figure 2).

### Comparison of Safety in Choledocholithiasis Patients at Different Age Groups

**Figure 3.**
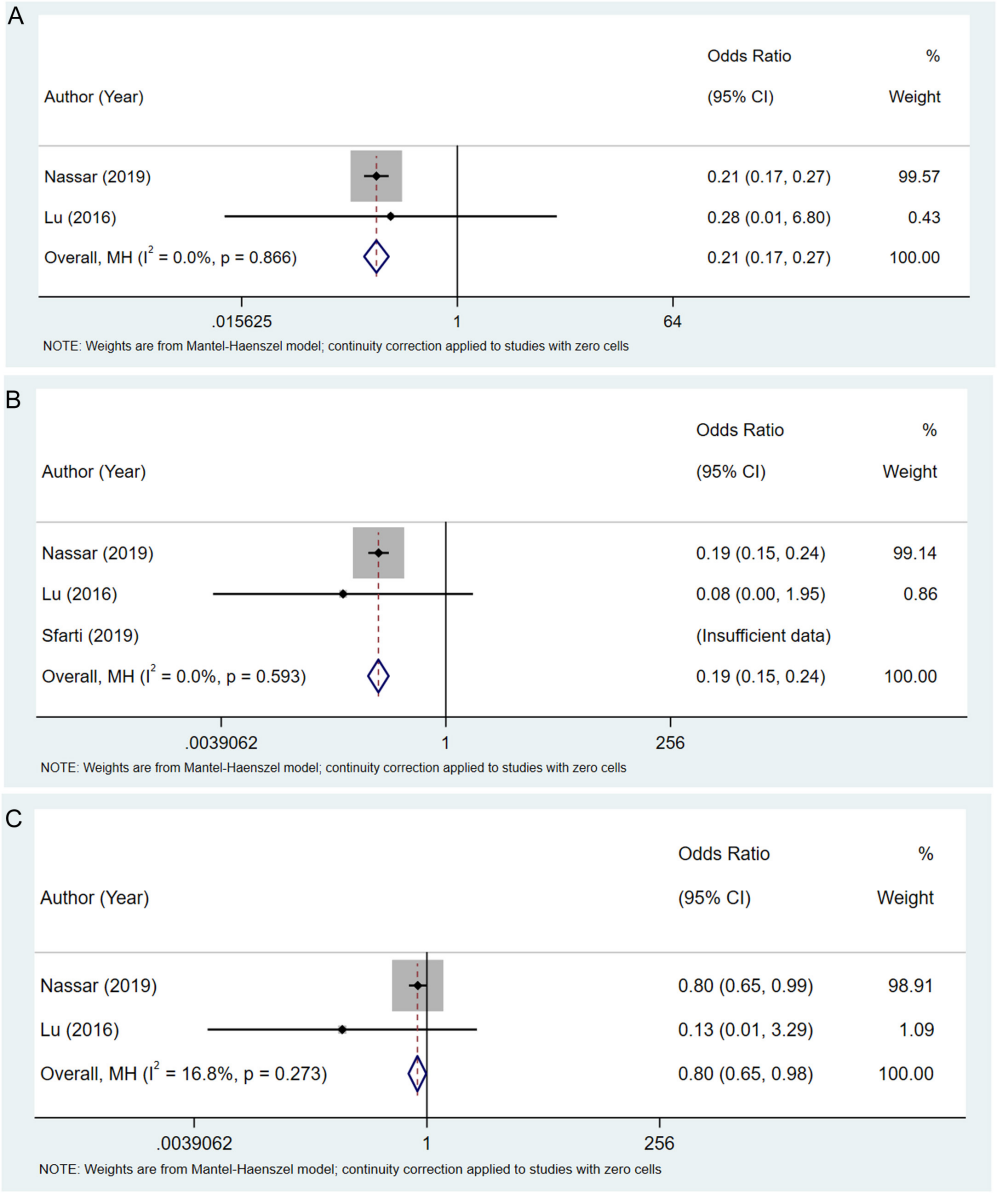
Forest plots regarding mortality between age <65 vs. age ≥65 (A), between age <65 vs. age ≥80 (B), and between the age range 65-80 vs. age ≥80 (C).

Table 2 shows that the odds of mortality for young patients (age <65 years) were lower than for patients aged ≥65 years (OR = 0.21, 95% CI: 0.17-0.27) (Figure 3A). Compared to the age ≥80 years group, the age <65 years and the age range 65-80 years groups had lower odds of mortality (OR = 0.19, 95% CI: 0.15-0.24; OR = 0.80, 95% CI: 0.65-0.98) (Figure 3B and C). Overall complications showed no statistical significance in choledocholithiasis patients at different age groups (both *P* > .05). Compared to the age ≥80 years group, higher odds of pancreatitis were found in the age <65 years group (OR = 5.08, 95% CI: 1.59-16.25). The odds of pneumonia were lower in general old patients (age range 65-90 years group) than in extremely old patients (age ≥90 years, OR = 0.16, 95% CI: 0.06-0.41).

### Sensitivity Analysis

Sensitivity analysis was carried out by removing each individual study from the meta-analysis, and no significant change was found in the results. This indicated the robustness of the results (Table 2).

## Discussion

In this meta-analysis, we found that complete stone clearance in generally old patients was higher than in extremely old patients. The mortality of young and generally old patients was lower than in extremely old patients. The odds of pancreatitis were higher in young patients compared to extremely old patients, while the odds of pneumonia were lower in general old patients compared to extremely old patients.

Life expectancy has been increasing worldwide in the past 2-3 decades, resulting in a steady increase in the number of elderly people.^8,11,12^ As age increases, the risk of choledocholithiasis also increases.^11,12^ It is evident that the demand for ERCP treatment for choledocholithiasis depends on age.^11,12^ Several studies have indicated that the efficacy and safety of ERCP for choledocholithiasis are comparable between elderly patients (≥65 years) and younger ones (<65 years).^8,11^ Most studies defined the elderly as patients aged ≥65 years, while the physical condition of people in their 60s is significantly different from that in eighties.^9^ Some studies have evaluated the efficacy and safety of ERCP in elderly patients, with a cut-off age of 80-85 years.^4,13^ Iida et al^4^ found that the complete removal rate of bile duct stones in patients <85 years was slightly higher than in those ≥85 years. Sugiyama and Atomi^14^ found complete stone clearance in 86% of patients aged ≥90 years and in 95% of patients <90 years. Our meta-analysis assessed complete stone clearance in different age groups and found that stone clearance was lower in extremely old patients compared to general old patients. This may be explained by the fact that extremely old patients had more severe conditions, poorer performance, and larger stones compared to general old patients; therefore, the complete stone clearance was significantly lower than that of general old patients.^15^

Previous studies have reported no difference in complication rate after ERCP between elderly patients and younger patients.^16,17^ Similarly, our meta-analysis found no statistical significance in the overall complications between young, generally old, and extremely old patients. Pancreatitis is the most common complication after ERCP, with a prevalence of 1.3%-8%.^11,18^ Interestingly, a study by Finkelmeier et al^19^ found the incidence of pancreatitis was lower in extremely old patients than in general old patients (0.9% vs. 4%) and young patients (0.9% vs. 5.4%). Han et al^11^ found that post-ERCP pancreatitis occurred in 1.3% of extremely old patients compared to 2.9% of younger patients. Therefore, some researchers considered that age was a protective factor for the development of post-ERCP pancreatitis.^20^ Consistent with previous studies, our meta-analysis displayed that the odds of post-ERCP pancreatitis were lower in extremely old patients than in young patients. There were some explanations for this finding. Extremely old patients may have a lower response to pancreatic trauma during surgery. This may be associated with anatomical changes that occur in the pancreas with age.^21^ Pancreatic atrophy occurs with age, and its weight may decrease from the normal 60-100 g to 40 g or less by the age of 85 years.^20,21^ In addition, histological changes in the pancreas can be observed in most elderly patients, including ductal epithelial cell proliferation with stratified squamous epithelium replacing normal ductal epithelium.^20^ Functionally, a decrease in pancreatic enzyme levels was found in the duodenal aspirates of elderly patients.^20^ Evidence has shown that age is an important factor in the occurrence of postoperative pneumonia.^22,23^ In addition, extremely old patients with severe respiratory system disorders may not be recommended to undergo ERCP.^24^ In this meta-analysis, we also found that the odds of pneumonia were higher in extremely old patients than in general old patients.

Age has been reported as an important influencing factor for death after ERCP.^25^ Nassar et al^25^ have found that the death rate post-ERCP increased with age, with 0.49% for patients aged less than 60 years, 1.59% for patients aged from 60 to 69 years, and 2.53% for patients aged more than 80 years. Consistently, in this meta-analysis, we found that the mortality of extremely old patients was higher than that of young and general old patients. This may be caused by the age-related natural decline in immune status, which is the main predisposing factor leading to increased mortality with age.^26^ In addition, the extremely old patients often suffer from chronic diseases and poor physical condition, which may increase postoperative mortality.^15^

To address the challenges associated with ERCP in older patients, multiple strategies can be implemented to improve the safety and efficacy of ERCP. First, careful preoperative evaluation is essential to improve stone clearance and reduce complications. In older patients, stones may be more difficult to remove due to anatomical and physiological changes.^15^ The use of ancillary techniques, such as extracorporeal shock wave lithotripsy, may improve stone clearance.^27^ Multidisciplinary team approach, including anesthetists and geriatricians, may enhance perioperative care and reduce postoperative complications such as pneumonia. Additionally, regular follow-up and customized management strategies for recurrent stones are necessary. Finally, in patients where ERCP is considered high risk, alternative treatment options should be carefully considered to minimize procedural complications. Percutaneous transhepatic stone removal can be an alternative treatment when ERCP is contraindicated or is not feasible.^28^ Endoscopic ultrasound-guided biliopancreatic drainage is also an effective percutaneous drainage modality and is increasingly becoming an important alternative to ERCP when it fails.^29^ Surgical interventions, including laparoscopic CBD exploration, offer a viable option for stone removal.^30^ Ultimately, treatment strategies must be tailored to each patient’s risk profile to ensure optimal outcomes.

This meta-analysis explores the efficacy and safety of ERCP in choledocholithiasis patients at different age groups. Results show that the complete stone clearance was lower and mortality was higher in extremely old patients undergoing ERCP compared to general old or younger patients, which may provide a reference for the use of ERCP in different age groups. However, there are some limitations in this meta-analysis. First, prospective studies are lacking in this meta-analysis. The results should be cautiously interpreted, and prospective studies are needed to verify our findings in the future. Second, included studies did not adequately report informative data on anesthesia complications, history of chronic disease, and reasons for failed intubation, which may also be a source of heterogeneity. Extremely old patients often suffer from chronic diseases, which may be one of the reasons for the increased mortality. The heterogeneity in the results regarding complete stone clearance is relatively high, which may be caused by the different stone removal techniques used in different studies. However, due to the limitation of included studies, subgroup analysis is unable to be further performed based on comorbidities and different stone removal techniques. Third, the number of included studies for some outcomes is small, which may affect the robustness of research results.

This meta-analysis found that extremely old patients had lower complete stone clearance and higher mortality after ERCP. The odds of post-ERCP pancreatitis were lower and pneumonia was higher in extremely old patients. Our findings indicated that ERCP should be cautiously used for extremely old patients with choledocholithiasis, and postoperative complications need to be monitored. In the future, more studies are needed to further verify our findings.

## Availability of Data and Materials:

The data that support the findings of this study are available on request from the corresponding author.

## Supplementary Materials

Supplementary Material

## Figures and Tables

**Table 1.** The Characteristics of Included Studies

Author	Year	Country	Study Design	Population	Age	Sample Size (n)	Sex (Male/Female)	Periampullary Diverticulum (n)	Antithrombotic Therapy (n)	Diameter of Stone (mm)	Cardiovascular Disease	ES	Outcomes	Quality Assessment
Nassar	2019	America	Cohort	Choledocholithiasis	<60	17 357		NA	NA	NA	NA	NA	In-hospital mortality	7
					70-79	7541		NA	NA	NA	NA	NA		
					≥80	8118		NA	NA	NA	NA	NA		
Lu	2016	China	Cohort	Choledocholithiasis	45-59	474	258/216	97	NA	8 (6-10)^^^	NA	362	In-hospital mortality, overall complications, pancreatitis, biliary infection, hemorrhage, perforation, complete stone removal	6
					68-77	281	159/122	101	NA	10 (8-12)^^^	NA	197		
					81-86.5	113	62/51	45	NA	10 (8-12)^^^	NA	89		
Saito	2019	Japan	Cohort	Choledocholithiasis	75-89	569	285/284	175	197	7.9 (2-25)^*^	101	382	In-hospital mortality, pancreatitis, acute cholangitis/cholecystitis, bleeding, perforation, pneumonia, heart failure, complete atrioventricular, cerebral infarction, respiratory dysfunction, complete stone removal	6
					≥90	126	39/87	46	33	9.4 (3-30)^*^	24	84		
Sfarti	2019	Europe	Case–control	Choledocholithiasis	51-60	260		19	NA	NA	NA	NA	Pancreatitis, bleeding, cholangitis, complete stone removal	7
					≥80	222		28	NA	NA	NA	NA		
Sugiyama	2000	Japan	Cohort	Choledocholithiasis	70-89	381	176/205	160	NA	≥15, 94	61	375	In-hospital mortality, overall complications, pancreatitis, bleeding, cholangitis, acute cholecystitis, hepatic failure, basket impaction, complete clearance	7
					90-96	22	10/12	13	NA	≥15, 11	8	22		
Jalal	2023	UK	Cohort	Choledocholithiasis	78 ± 7^#^	1370	616/754	181	360	>10, 258	922	983	30-day death, overall complications, pancreatitis, bleeding, perforation, cholangitis/cholecystitis, cardiovascular events	6
					93 ± 3^#^	128	52/76	26	48	>10, 38	102	94		
Christoforidis	2008	Europe	Cohort	Choledocholithiasis	79 (75-89) ^*^	272	118/154	79	NA	>10, 105	44	272	In-hospital mortality, overall complications, cholangitis, pancreatitis, acute cholecystitis, basket impaction, complete clearance	6
					92 (90-99) ^*^	33	11/22	15	NA	>10, 28	12	33		
Hakuta	2024	Japan	Retrospective cohort study	Choledocholithiasis	<65 (n = 68); ≥65, (n = 63)	131	68	NA	NA	9 (6-14)^^^	NA	NA	Complete clearance	7
Gustafsson	2023	Sweden	Retrospective cohort study	Choledocholithiasis	<65, (n = 873); ≥65, (n = 699)	1605	915	NA	NA	NA	NA	NA	Complications	5
Wu	2023	China	Retrospective cohort study	Choledocholithiasis	<65, (n = 491); ≥65, (n = 201)	692	340	140	NA	<15, 328; ≥15, 215	48	NA	Recurrence of choledocholithiasis, complete clearance	6

ES, endoscopic sphincterotomy; n, number; NA, not available.

#Mean ± SD. *Median (minimum, maximum). ^Median [interquartile range (IQR) 25th–75th percentile].

**Table 2.** Comparison of Efficiency and Safety of ERCP in Choledocholithiasis Patients at Different Age Groups

Outcome	Indicator	Number of Studies	OR (95% CI)	*P*	*I*^2^ (%)
Complete stone clearance					
<65 vs. ≥65	Overall	2	0.762 (0.194, 2.996)	.697	87.9
	Sensitive analysis		0.762 (0.194, 2.996)		
<65 vs. ≥80	Overall	2	0.881 (0.623, 1.245)	.474	34.4
	Sensitive analysis		0.881 (0.623, 1.245)		
65-80 vs. ≥80	Overall	1	0.877 (0.411, 1.871)	.735	
65-90 vs. ≥90	Overall	3	7.601 (1.890, 30.566)	.004	86.3
	Sensitive analysis		7.601 (1.890, 30.566)		
Mortality					
<65 vs. ≥65	Overall	2	0.210 (0.166, 0.266)	<.001	0.0
	Sensitive analysis		0.210 (0.166, 0.266)		
<65 vs. ≥80	Overall	3	0.189 (0.147, 0.244)	<.001	0.0
	Sensitive analysis		0.189 (0.147, 0.244)		
65-80 vs. ≥80	Overall	2	0.797 (0.646, 0.984)	.035	16.8
	Sensitive analysis		0.797 (0.646, 0.984)		
65-90 vs. ≥90	Overall	4	0.527 (0.209, 1.330)	.175	0.0
	Sensitive analysis		0.527 (0.209, 1.330)		
Recurrence of choledocholithiasis					
<65 vs. ≥65	Overall	1	0.539 (0.357, 0.812)	.003	
Overall complications					
<65 vs. ≥65	Overall	2	1.153 (0.906, 1.468)	.247	0.0
	Sensitive analysis		1.153 (0.906, 1.468)		
<65 vs. ≥80	Overall	2	0.553 (0.028, 10.847)	.696	95.6
	Sensitive analysis		0.553 (0.028, 10.847)		
65-80 vs. ≥80	Overall	1	2.202 (0.824, 5.887)	.116	
65-90 vs. ≥90	Overall	4	0.705 (0.457, 1.087)	.114	0.0
	Sensitive analysis		0.705 (0.457, 1.087)		
Pancreatitis					
<65 vs. ≥65	Overall	1	1.869 (0.874, 3.996)	.107	
<65 vs. ≥80	Overall	2	5.078 (1.586, 16.252)	.006	
	Sensitive analysis		5.078 (1.586, 16.252)		
65-80 vs. ≥80	Overall	1	8.779 (0.510, 151.085)	.135	
65-90 vs. ≥90	Overall	4	1.809 (0.687, 4.767)	.230	
	Sensitive analysis		1.809 (0.687, 4.767)		
Perforation					
<65 vs. ≥65	Overall	1	2.499 (0.102, 61.527)	.575	
<65 vs. ≥80	Overall	2	0.719 (0.029, 17.768)	.840	0.0
65-90 vs. ≥90	Overall	3	0.795 (0.140, 4.508)	.796	
	Sensitive analysis		0.795 (0.140, 4.508)		0.0
Biliary infection					
<65 vs. ≥65	Overall	2	0.931 (0.605, 1.433)	.745	
	Sensitive analysis		0.931 (0.605, 1.433)		
<65 vs. ≥80	Overall	2	3.119 (0.845, 11.518)	.088	
	Sensitive analysis		3.119 (0.845, 11.518)		0.0
65-80 vs. ≥80	Overall	1	2.048 (0.442, 9.497)	.360	
65-90 vs. ≥90	Overall	2	1.432 (0.378, 5.420)	.597	
	Sensitive analysis		1.432 (0.378, 5.420)		29.3
Bleeding					
<65 vs. ≥65	Overall	1	1.461 (0.425, 5.029)	.547	
<65 vs. ≥80	Overall	2	4.963 (0.974, 25.295)	.054	0.0
	Sensitive analysis		4.963 (0.974, 25.295)		
65-80 vs. ≥80	Overall	1	1.209 (0.124, 11.743)	.870	
65-90 vs. ≥90	Overall	4	0.419 (0.171, 1.027)	.057	0.0
	Sensitive analysis		0.419 (0.171, 1.027)		
Pneumonia					
65-90 vs. ≥90	Overall	2	0.158 (0.061, 0.412)	<.001	0.0
	Sensitive analysis		0.158 (0.061, 0.412)		

ERCP, endoscopic retrograde cholangiopancreatography; OR, odds ratio; vs., versus.

## Supplementary File 1

The search strategies used were listed as the following: cholangiopancreatography, endoscopic retrograde; retrograde cholangiopancreatography, endoscopic; cholangiopancreatographies, endoscopic retrograde; endoscopic retrograde cholangiopancreatographies; retrograde cholangiopancreatographies, endoscopic; endoscopic retrograde cholangiopancreatography; ERCP; cholelithiasis; cholelithiases; gallstone disease; gallstone diseases; cholecystolithiasis; choledocholithiasis; common bile duct; gallstones; gallstone; gall stones; biliary calculi; calculi, biliary; gall stone; common bile duct calculi; biliary calculi, common bile duct; gallstones, common bile duct; common bile duct gall stone; common bile duct gallstones; gall stones, common bile duct; common bile duct gallstone; common bile duct gall stones; age; aged; elderly; old.
