# Supplementary material for: Comparison of Efficacy and Safety of Endoscopic Retrograde Cholangiopancreatography in Choledocholithiasis Patients at Different Age Groups: A Meta-Analysis
Source: Turk J Gastroenterol. 2025 Jan 13;36(6):398–407. doi: 10.5152/tjg.2025.24003 (PMC12147330; doi:10.5152/tjg.2025.24003)
Supplement: Supplementary Material [file supplementary_material.pdf]

Supplementary File 1

The search strategies used were listed as the following: cholangiopancreatography, endoscopic retrograde; retrograde cholangiopancreatography, endoscopic; cholangiopancreatographies, endoscopic retrograde; endoscopic retrograde cholangiopancreatographies; retrograde cholangiopancreatographies, endoscopic; endoscopic retrograde cholangiopancreatography; ERCP; cholelithiasis; cholelithiases; gallstone disease; gallstone diseases; cholecystolithiasis; choledocholithiasis; common bile duct; gallstones; gallstone; gall stones; biliary calculi; calculi, biliary; gall stone; common bile duct calculi; biliary calculi, common bile duct; gallstones, common bile duct; common bile duct gall stone; common bile duct gallstones; gall stones, common bile duct; common bile duct gallstone; common bile duct gall stones; age; aged; elderly; old.
